# The Effect of Training Experience and Leg Dominance on the Prevalence of Asymptomatic Intraarticular Changes of the Knee Joints in Adult Professional Male Soccer Players

**DOI:** 10.1186/s40798-020-00248-9

**Published:** 2020-04-19

**Authors:** Eduard Nikolayevich Bezuglov, Vladimir Yurevich Khaitin, Anastasiya Vladimirovna Lyubushkina, Artemii Mikhailovich Lazarev, Artem Valerievich Gorinov, Elena Yurevna Sivakova, Elizaveta Ilinichna Rumiantseva, Alexey Vladimirovich Lychagin

**Affiliations:** 1 Department of Sport Medicine and Medical Rehabilitation, Sechenov First Moscow State Medical University (Sechenov University), Moscow, Russian Federation; 2 Federal Research and Clinical Center of Sports Medicine and Rehabilitation of Federal Medical Biological Agency, Moscow, Russia; 3 High Performance Sport Laboratory, Moscow Witte University, Moscow, Russia; 4 Department of Sport Medicine, Pavlov First Saint-Petersburg State Medical University, Saint-Petersburg, Russia; 5 FC Zenit Saint-Petersburg, Saint-Petersburg, Russian Federation; 6 «Smart Recovery» Clinic, Moscow, Russian Federation; 7 Clinical Diagnostic Center “Medsi” on Belorusskaya, Moscow, Russian Federation; 8 Federal State Budgetary Institution “Clinical Hospital”, Moscow, Russian Federation; 9 Faculty of Fundamental Medicine, Lomonosov Moscow State University, Moscow, Russian Federation; 10 Department of Traumatology and Orthopaedic Surgery, Sechenov First Moscow State Medical University (Sechenov University), Moscow, Russian Federation

**Keywords:** Professional soccer players, Asymptomatic findings in knee, Magnetic resonance imaging, Meniscus tears, Cartilage injury

## Abstract

**Background:**

Currently, no data is available regarding the association between professional experience or limb dominance and the prevalence of asymptomatic knee joint lesions in adult professional male soccer players.

**Hypothesis:**

The prevalence of the accumulated changes increases with training experience. This is especially true for the dominant leg, which is involved in a large proportion of the athletes’ movements.

**Study Design:**

Level 2 cross-sectional cohort study

**Methods:**

MRI was used to assess the condition of 94 knee joints in 47 adult professional male soccer players (mean age 25.7 ± 4.6 years, BMI 22.8 ± 1.4). Previous surgery on joints was an exclusion criterion. No football player had knee injuries (including fresh bruises) for at least 3 months before the examination. All the scans were performed using a 1.5T MRI scanner and a slice thickness of 3 mm. The images were blindly analyzed by two experienced radiologists. We analyzed all the three compartments of the knee joint. We consider a chondral lesion already from grade I in modified Noyes and Stabler classification system. To assess the influence of soccer training experience, all players were divided into two groups: group 1 formed from players with less than 20 years of experience and group 2 with more than 20 years of experience.

**Results:**

One hundred percent of the soccer players had at least one chondral and meniscal lesion.

In both legs, the posterior horn of the medial meniscus (95.6%) was the most frequent site of injury. Most of the injuries were classified as grade II injuries (73.3% for the dominant and 75.6% for the non-dominant leg).

Experience and age of the athletes significantly increased the probability of subcortical bone lesions. They were significantly positively correlated with the grades of patellar lesions and lesions of the patellar surface of the femur and significantly negatively correlated with the grades of lesions of posterior horn of lateral meniscus and anterior horn of medial meniscus.

No statistically significant differences in the prevalence and grades of cartilage and meniscal lesions in the dominant and non-dominant limb were observed.

**Conclusion:**

Soccer practice is associated with the increased prevalence of asymptomatic chondral and meniscal lesions.

The probability of subcortical bone lesions significantly increases with training experience and age. These factors are also positively correlated with the grades of patellar lesions and lesions of the patellar surface of the femur.

The prevalence and grade of asymptomatic chondral and meniscal lesions is independent of leg dominance.

## Key Points

- Soccer practice is associated with the increased prevalence of asymptomatic intraarticular changes of the knee.
- One hundred percent of the soccer players had at least one chondral and meniscal lesion.
- The prevalence of asymptomatic chondral and meniscal lesions is independent of leg dominance.

## Introduction

Currently, multiple articles on the prevalence of asymptomatic changes in the knee joints of different groups of individuals, including athletes, have been published [1–3].

Studies conducted among non-athletes have shown that the number of various intraarticular changes in the knee joints increases with age.

For example, the prevalence of asymptomatic meniscus damage in people younger than 45 years old is 13–37%, increasing to 56% in people above 70 years old [4].

The available data confirm that various intraarticular lesions of this type are more prevalent in professional athletes who participate in different types of sports compared to non-athletes [4–9].

According to a review by Flanigan et al., the mean prevalence of full-thickness chondral defects in a group of 931 athletes (mean age 33 years) was 36%, with 14% of the cases being asymptomatic. In 47% of cases, meniscal lesions of various grades were observed [10].

At the same time, Soder et al., who analyzed young soccer players and swimmers aged 14–15 years, found that neither the athletes nor the control group had any meniscal lesions or significant chondral defects [11, 12].

In a similar study by Mattioti et al., the prevalence of meniscal and chondral lesions in a cohort of young soccer players aged 14 to 17 years was low (10.9% and 8.7%, respectively) [13].

Regular soccer training results in the increased prevalence of adverse changes in the large joints of the lower extremities due to constant microtraumatization [14, 15].

Knee joint osteoarthritis is thus more prevalent in former soccer players, even those who have never undergone surgery and have never received knee injuries, compared to the general population, which may result in the decreased quality of life [16, 17].

Soccer is one of the few games where the lower extremities are constantly used in every component of the game. One of the legs, called the dominant leg, is the most often used for the technical elements such as ball handling, passing, and hitting the ball [18–21]. This results in an uneven development of motor patterns, which can in turn lead to strength imbalance, which increases with experience, and to traumas [20, 22].

Several studies on the trends associated with leg dominance in soccer players exist. The data obtained in these studies are contradictory.

Fousekis et al. demonstrated that lower limbs in soccer players may develop asymmetrically depending on the limb dominance and that this asymmetry increases with training experience. The dominance of the limb and training experience can influence the pre-existing anatomical and functional asymmetry and lead to injuries [23].

The study by Kearns et al. shows that predominant use of one of the lower limbs is associated with an increase in muscle thickness of the dominant leg in young football players [24].

Magnetic resonance imaging (MRI), which is an accurate tool for the detection of intraarticular cartilage lesions, meniscal tears, ligament ruptures, and bone marrow edema, is currently used to assess the condition of intraarticular structures both in acute trauma and various regular check-ups [25–27].

To achieve better results, 1.5–3T MRI scanners are used for the analysis of intraarticular structures, as they provide a significantly higher sensitivity, specificity, and precision in detection of chondral lesions compared to low-field MRI scanners [28].

The prevalence of asymptomatic lesions of intraarticular structures of knee joint in the general population is relatively high and is associated with age. However, there is no evidence linking the prevalence of these lesions to the age of the athlete or their training experience.

Despite the fact that soccer is one of the most popular sports in the world, we could not find any research regarding the association between the prevalence of asymptomatic knee joint lesions in soccer players and either their experience or the dominance of a specific limb.

In this regard, studying the association between the prevalence of asymptomatic lesions of intraarticular structures and experience, age, and limb dominance in adult professional soccer players seems to be of immediate interest. This knowledge will allow to objectively assess the impact of external factors such as training experience and limb dominance on the prevalence of various intraarticular lesions, as well as develop prevention programs for different age groups to help combat joint injuries and avoid incorrect interpretation of MRI data obtained in soccer players of various ages with acute injuries.

## Methods

All participants signed a written informed consent. The study was performed in accordance with the standards of ethics outlined in the Declaration of Helsinki. The conduction of this study at this research institution was also approved by the local ethics committee of the Sechenov First Moscow State Medical University [extract from the Protocol 11–19 of the special meeting of the Local Ethics Committee on 25.07.2019].

### Patients

This controlled cohort study continued from December 2014 until January 2019. Forty-seven male professional elite soccer players who underwent medical examinations prior to signing a contract with the top Russian Premier League clubs were included (mean age 25.7 ± 4.6 years, BMI 22.8 ± 1.4).

To assess the influence of soccer training experience, all players were divided into two groups, with group 1 formed from players with less than 20 years of experience and group 2 formed from players with more than 20 years of experience. By term “experience” meant that all the players who participated in the study began to play football at the age of 6–7 years. Thus, the lag point was the same for all athletes. All the players who participated in the study were only ever regularly involved in one sport—football.

1.5T MRI scanner was used to analyze the 94 knee joints of these players.

All the participants of the study were members of adult or youth national teams of their countries and played at least 80 matches in the professional leagues of their countries during their careers.

The criteria for inclusion in the study were as follows:

- age of the athlete, 18 years and older;
- no knee joint complaints at the time of examination;
- no medical history of knee joint surgery or any other joint surgery;
- no medical history of intraarticular punctures of the knee joint;
- signing a contract with the club based on the results of the examination.

The criteria for exclusion in the study were as follows:

- prior medical history of knee joint surgery or any other joint surgery;
- age 8 years and older at the beginning of their systematic soccer training;
- participation in a soccer match 5 days or less prior to their MRI scan;
- a medical history of knee joint trauma or surgery in the 12 months following the examination
- the goalkeeper position on the field.

The information provided by the players was verified using the medical records provided by the club medical staff and the data obtained from the website transfermarket.de.

### Magnetic Resonance Tomography

All imaging were conducted using a 1.5T MRI scanner (Philips Ingenia, Amsterdam, the Netherlands and Siemens Magnetom, Munich, Germany). Images in sagittal, axial, and coronal planes were obtained for analysis using standard pulse sequences (short tau inversion recovery (STIR) images TR/TE) and fast spin-echo T1-weighted images (TR/TE) in sagittal plane. Proton density (PD) weighted images were used for the interpretation of BMO (bone marrow oedema). The layer thickness was 3 mm.

The images were evaluated to detect the presence or absence of abnormalities. The following abnormalities were evaluated: joint effusion, bone marrow edema, meniscus, and chondral abnormalities.

A total of six articular surfaces were evaluated, including those of the patella, medial femoral condyle, lateral femoral condyle, medial tibial condyle, lateral condyle of tibia, and patellar surface of femoral bone. Articular cartilages were graded based on a modification of the Noyes and Stabler classification system [29]:

- Grade 0, normal thickness and signal;
- Grade I, normal thickness but an altered signal;
- Grade II, superficial partial-thickness cartilage defect affecting less than 50% of the total cartilage thickness;
- Grade III, deep partial-thickness cartilage defect affecting more than 50% of the total cartilage thickness;
- Grade IV, full-thickness chondral defect with exposure of subchondral bone.

The degree of meniscus damage was graded separately according to the methods described by Stoller et al. [30]:

- Grade 0, normal signal;
- Grade I, one or several punctuate signal intensities that do not reach the surface of the meniscus;
- Grade II, linear signal intensity that does not reach the surface of the meniscus;
- Grade III, signal intensity that reaches the surface of the meniscus.

Also, we evaluated the presence of intraarticular osteophytes.

Synovitis was interpreted as the presence of more than 5 mL of synovial fluid in the suprapatellar bursa [31].

The presence of BMO was estimated based on a low signal on T1-weighted images and a high signal intensity on PD (proton density) weighted images.

### Image Analysis

All images were obtained using eFilm Workstation (IBM, Armonk, USA) and saved for later analysis. Two radiologists with at least 7 years of experience of working with athletes evaluated all images independently of each other. Neither of them knew the age, type, and level of physical activity of patients and whether the left and right legs belonged to one person. If there was disagreement between the radiologists, the final decision was made by a third independent radiologist.

### Statistical Analysis

The data were stored in a Microsoft Excel spreadsheet and analyzed using the SPSS statistical package, version 23.0 (IBM, Armonk, USA). The results were considered statistically significant at *p* ≤ 0.05. Distribution normality was assessed using the Kolmogorov-Smirnov test. Student’s independent samples *t*-test was used to compare the results of the two groups. Logistic regression was used to analyze the association between age, training experience, and various lesions. Correlation between age, experience, and frequency of lesions was assessed using Spearman’s rank correlation coefficient, and the association between leg dominance and lesion frequency was assessed using Pearson’s chi-squared test.

## Results

The MRI data of the right and left knee joints of 47 soccer players (94 joints in total) were analyzed. Dominant leg was defined as one that the player uses to kick the ball with the maximal strength and dexterity. Right leg was dominant in 79% (37) of the soccer players, left leg—in 17% (8) of the players. Four percent (2) of the players had no distinct dominant leg, and their MRI data were used in both categories.

One hundred percent of the soccer players had at least one chondral and meniscal lesion.

The athletes were divided into two groups based on their age, with athletes aged 26 years and younger forming group 1 and athletes aged 27 and older forming group 2. No statistically significant difference in weight, height, and body mass index between the groups was observed (Table 1).

**Table 1:** Height, weight, and BMI of the athletes in groups 1 and 2

		*N*	Mean (SD)	*p* value
Height, cm	Group 1	21	181.9 (6.2)	0.26
	Group 2	26	184.3 (7.8)	
Weight, kg	Group 1	21	75.8 (6.5)	0.49
	Group 2	26	77.0 (6.0)	
BMI	Group 1	21	22.9 (1.4)	0.62
	Group 2	26	22.7 (1.4)	

Logistic regression was used to perform a pairwise assessment of associations between players’ experience or age and the presence of osteophytes, synovitis, bone marrow edema, or subcortical bone lesions (Table 2). According to the analysis, there was a statistically significant association between experience and age of athletes and the probability of subcortical bone lesions.

**Table 2:** The association between experience or age and lesions of the knee joint

Lesion of the knee joint	Experience	Age
Osteophytes	*p* = 0.19; OR = 1.19	*p* = 0.30; OR = 1.14
Synovitis	*p* = 0.19; OR = 1.09	*p* = 0.25; OR = 1.08
Bone marrow edema	*p* = 0.13; OR = 1.13	*p* = 0.131; OR = 1.13
Subcortical bone lesions	*p* = 0.06	*p* = 0.005
	OR = 1.231	OR = 1.240
	95% CI 1.061–1.427	95% CI 1.067–1.441

*p* one-sided significance of the analyzed outcome variable

Spearman’s rank correlation coefficient was calculated to analyze the correlation between experience or age and the MRI grade of articular lesions. The results are presented in Table 3. The experience and age of the athletes were significantly positively correlated with the grades of patellar cartilage lesions and lesions of the patellar surface of the femur. They were also significantly negatively correlated with the grades of lesions of the posterior horn of the lateral meniscus and the anterior horn of the medial meniscus.

**Table 3:**
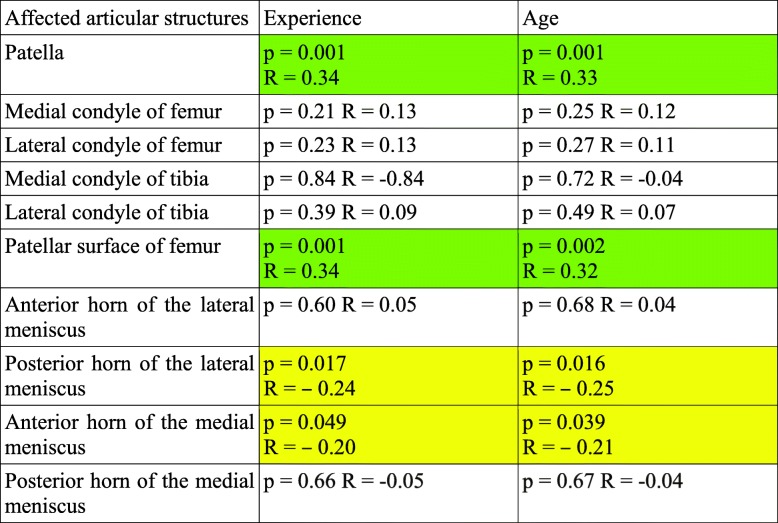
The association between age or experience and the affected articular structures

*p* two-sided significance of the correlation between experience or age and lesions of specific articular structures, *R* correlation coefficient (*p* < 0.05 if shown)

The prevalence of various lesions in the dominant and non-dominant limbs is presented in Table 4. It can be concluded that there is no significant difference between the prevalence of lesions in the dominant and non-dominant limbs. A statistically significant difference between the prevalence of the lesion in the dominant and non-dominant limb was only observed for grade 2 lesions of the lateral condyle of femur (*p* = 0.002). However, total prevalence of lesions at this site was independent of limb dominance (*p* = 0.07).

**Table 4:** The prevalence of specific lesions in the dominant and non-dominant limb

	Dominant limb (*n* = 47)	Non-dominant limb (*n* = 47)	*p*
Chondral lesions			
Grade 1 Pat, *n* (%)	10 (21)	12 (26)	0.63
Grade 2 Pat, *n* (%)	20 (43)	21 (45)	0.84
Grade 3 Pat, *n* (%)	5 (11)	4 (9)	0.73
Grade 1 MCF, *n* (%)	2 (4)	7 (15)	0.08
Grade 2 MCF, *n* (%)	17 (36)	17 (36)	>0.99
Grade 3 MCF, *n* (%)	19 (40)	15 (32)	0.39
Grade 4 MCF, *n* (%)	3 (6)	0 (0)	0.08
Grade 1 LCF, *n* (%)	7 (15)	7 (15)	> 0.99
Grade 2 LCF, *n* (%)	23 (49)	17 (36)	0.21
Grade 3 LCF, *n* (%)	3 (7)	15 (32)	0.002
Grade 4 LCF, *n* (%)	1 (2)	2 (4)	0.56
Grade 1 MCT, *n* (%)	8 (17)	5 (11)	0.37
Grade 2 MCT, *n* (%)	26 (55)	28 (60)	0.68
Grade 3 MCT, *n* (%)	5 (11)	5 (11)	> 0.99
Grade 4 MCT, *n* (%)	1 (2)	0 (0)	> 0.99
Grade 1 LCT, *n* (%)	7 (15)	13 (28)	0.13
Grade 2 LCT, *n* (%)	23 (49)	22 (47)	0.84
Grade 3 LCT, *n* (%)	1 (2)	2 (4)	0.56
Grade 4 LCT, *n* (%)	2 (4)	0 (0)	0.15
Meniscal lesions			
Grade 1 AHLM, *n* (%)	6 (13)	5 (11)	0.75
Grade 2 AHLM, *n* (%)	20 (43)	17 (36)	0.53
Grade 3 AHLM, *n* (%)	2 (4)	1 (2)	0.56
Grade 1 PHLM, *n* (%)	6 (13)	5 (11)	0.75
Grade 2 PHLM, *n* (%)	22 (47)	24 (51)	0.68
Grade 3 PHLM, *n* (%)	1 (2)	0 (0)	> 0.99
Grade 1 AHMM, *n* (%)	6 (13)	9 (19)	0.4
Grade 2 AHMM, *n* (%)	20 (43)	21 (45)	0.84
Grade 3 AHMM, *n* (%)	1 (2)	0 (0)	> 0.99
Grade 1 PHMM, *n* (%)	6 (13)	5 (11)	0.75
Grade 2 PHMM, *n* (%)	34 (72)	35 (74)	0.82
Grade 3 PHMM, *n* (%)	3 (6)	4 (9)	0.69
Osteoarthritis, *n* (%)	2 (4)	2 (4)	> 0.99
Synovitis, *n* (%)	5 (11)	10 (22)	0.16
Bone marrow edema, *n* (%)	6 (13)	4 (9)	0.53
Subcortical bone lesion, *n* (%)	8 (18)	9 (20)	0.97

*Pat* patella, *MCF* medial condyle of femur, *LCF* lateral condyle of femur, *MCT* medial condyle of tibia, *LCT* lateral condyle of tibia, *AHLM/PHLM* anterior/posterior horn of the lateral meniscus, *AHMM/PHMM* anterior/posterior horn of the medial meniscus

## Discussion

In the general population of people younger than 40 years of age without complaints, without surgery, and serious injuries in anamnesis, osteoarthritis occurs in 4–14%, cartilage damage in 11% and meniscus damage in 4% of cases. In the age group over 40 years old, osteoarthritis was detected in 19–43% and damage of the cartilage and menisci in 43% and 19% of cases, respectively [32].

According to this study, soccer is associated with an increased prevalence of asymptomatic chondral and meniscal lesions compared to the general population.

Association of systematic football training with the condition of intraarticular structures was also the subject of study by Matiotti et al. The authors have analyzed the 3-T MRI scans of the knee joints of 23 Brazilian junior soccer players. The control group was composed of volunteers comparable by age and body weight whose engagement in any physical activity did not exceed 100 min per week. Medical history of knee trauma or knee surgery was selected as an exclusion criterion. In the soccer players, 67.4% of the joints had at least one change detected by MRI, as opposed to 48.4% in the control group. Bone marrow edema was the most prevalent finding (41.3% and 7.3% of the joints in case and control groups, respectively). This corresponds well to the results obtained by Soder et al. Synovitis was detected in 19% of the joints in the case group, implicit cartilage lesions—in 8.7% of the joints, and lesions of the posterior horn of the medial meniscus—in 10.8% of the joints. Joint effusion was the most prevalent finding in the control group (19.4% of the joints). Hoffa’s fat pad edema was detected in 9.8% of the joints, and no cartilage or meniscal lesions were found. The increase in the incidence of cartilage and meniscal lesions in the soccer players analyzed by Matiotti et al. can be caused by either the increased age of the participants, which would result in the longer period of systematic soccer training, or the usage of 3-T MRI, which provides better sensitivity and specificity for the detection of cartilage lesions compared to the low field 0.35-T MRI [13].

A number of articles have been published analyzing basketball players, swimmers, long-distance runners, gymnasts, football players, and kangoo jumpers [5–8, 33, 34]. Notably, most of the studies concerning asymptomatic changes of the large joints primarily analyze amateurs [9, 13, 32, 35].

The article by Brunner et al. can be considered the first study on asymptomatic changes of the knee joints in athletes. Using MRI, the authors discovered that meniscal changes had developed in 50% of the basketball players who participated in the study [35].

Experience and age are significantly associated with the probability of subcortical bone lesions and are positively correlated with the grades of patellar cartilage lesions and lesions of the patellar surface of the femur.

The prevalence of asymptomatic chondral and meniscal lesions does not depend on the dominance of a particular limb. The most significant chondral lesions are, however, localized in the dominant leg.

Thus, our hypothesis is partially confirmed, as the association between lesion grade and experience was found. There is, however, no statistically significant association between lesion prevalence and limb dominance.

The results show that limb dominance does not affect the overall prevalence of chondral and meniscal lesions.

One of the previously published studies showed that ACL tears are positively correlated with limb dominance in male soccer players, while in female soccer players the non-dominant limb was more commonly affected. That study, however, included soccer players of various levels, and the overall proportion of professional players was low [36].

Similar results were obtained by Ruedl et al., who studied female skiers. This study, however, also included amateur athletes [37].

In another study, Greska et al. concluded that foot dominance did not negatively affect any known biomechanical risk factors for non-contact ACL tears [38].

At the same time, Ludwig et al. assessed the dynamic measures of lower limb joint angle at landing in soccer players of various levels. They observed a statistically significant difference between the limbs, with the non-dominant limb being more stable at landing [39]. This may present a significant risk factor for knee injury in the dominant leg in football players and corresponds to the data obtained by Brophy et al [36].

We could not find any data regarding the effect of lower limb dominance on the development of any intraarticular changes in professional soccer players.

Neither could we find any studies regarding the association between the prevalence of joint lesions and age or experience in professional soccer players. However, the analysis of the published data allows us to conclude that meniscal and chondral lesions may become more prevalent with experience. For example, Beals et al., having analyzed 14 studies on the prevalence of asymptomatic meniscal lesions in adult amateur and professional athletes (mean age 31.2 years), observed such lesions in 31.3% of the participants [40].

An even higher prevalence of asymptomatic meniscal lesions was reported by Flanigan et al. [10].

At the same time, the rate of such injuries in younger soccer players is minimal. All currently published studies have assessed the prevalence of lesions without accounting for the effect of age and experience of athletes.

The weaknesses of this study include the absence of a control group composed of the individuals from the general population, as well as the insufficient number of soccer players with dominant left foot, which prevented us from assessing the impact of limb dominance on the grade of intraarticular lesions.

Future research should focus on studying intraarticular lesions in large joints of the lower limb in a group of older soccer players with no anamnesis of trauma, as well as on comparing MRI data of professional soccer players of various ages with the data obtained from the athletes participating in other sports and from the members of the general population that will allow to objectively assess the impact of soccer on joint health, develop prevention programs, and adequately assess the MRI data in this group of athletes. That, in turn, will lead to a decrease in the number of unwarranted surgeries.

## Conclusion

Soccer training is associated with the increased prevalence of asymptomatic chondral and meniscal changes.

The probability of subcortical bone lesions significantly increases with training experience and age. These factors are also positively correlated with the grades of patellar lesions and lesions of the patellar surface of the femur.

The prevalence and grade of asymptomatic chondral and meniscal lesions is independent of leg dominance.

## Supplementary information

**Additional file 1:.** The effect of training experience and leg dominance on the prevalence of asymptomatic intraarticular changes of the knee joints in adult professional male soccer players.

## Data Availability

The datasets generated and analyzed during the current study are available as supplementary material, also from the corresponding author on reasonable request.

## Abbreviations

MRI: Magnetic resonance imaging
BMI: Body mass index
STIR: Short tau inversion recovery
PD: Proton density
BMO: Bone marrow oedema
MCF: Medial condyle of femur
LCF: Lateral condyle of femur
MCT: Medial condyle of tibia
LCT: Lateral condyle of tibia
AHLM: Anterior horn of the lateral meniscus
PHLM: Posterior horn of the lateral meniscus
AHMM: Anterior horn of the medial meniscus
PHMM: Posterior horn of the medial meniscus
ACL: Anterior cruciate ligament
